# Increasing Prevalence of Pediatric Metabolic Syndrome and Its Components among Arab Youth: A Time-Series Study from 2010–2019

**DOI:** 10.3390/children8121129

**Published:** 2021-12-03

**Authors:** Osama E. Amer, Shaun Sabico, Malak N. K. Khattak, Abdullah M. Alnaami, Naji J. Aljohani, Hanan Alfawaz, Abdulaziz AlHameidi, Nasser M. Al-Daghri

**Affiliations:** 1 Biochemistry Department, King Saud University College of Science, Riyadh 11451, Saudi Arabia; osamaemam@gmail.com (O.E.A.); eaglescout01@yahoo.com (S.S.); malaknawaz@yahoo.com (M.N.K.K.); aalnaami@yahoo.com (A.M.A.); 2 Obesity, Endocrine and Metabolic Center, King Fahad Medical City, Riyadh 11525, Saudi Arabia; najij@hotmail.com; 3 Department of Food Science and Nutrition, College of Food Science and Agriculture, King Saud University, Riyadh 11495, Saudi Arabia; halfawaz@ksu.edu.sa; 4 Saudi Diabetes Charity, Riyadh 12721, Saudi Arabia; alsukkary@gmail.com

**Keywords:** pediatric metabolic syndrome, Arab children, obesity, time-series

## Abstract

Background: Metabolic syndrome (MetS) is a cluster of cardiometabolic disorders, mostly studied in adults with certain ethnic groups, such as Arabs with higher predisposition and heritability. In this time-series study, we tried to determine the prevalence of MetS in the Arabic pediatric population from 2010–2019 to gauge the need to intervene in the younger population. Methods: A total of 7985 Saudi school children aged 12–18 coming from different sets of cohorts in different timepoints were included in this time-series studies (Year 2010, *n* = 2081, 877 boys and 1204 girls; Year 2015, *n* = 3360, 1625 boys and 1735 girls, Year 2019, *n* = 2544, 956 boys and 1588 girls). Anthropometrics were measured as well as fasting blood samples for the assessment of lipids and glucose. Screening for MetS was conducted using the pediatric definition obtained from the 2004 definition of de Ferranti et al. Results: In 2010, the prevalence of MetS was 11.8%, which increased sharply to 20.1% in 2015 and again in 2019 to 20.6%. Stratified according to sex, the increased prevalence of MetS was more evident in boys with only 7.8% in 2010 jumping to 25.3% in 2019. In girls, the increase in prevalence was modest with 14.8% in 2010 to 17.7% in 2019. In both boys and girls, the highest increase in prevalence among MetS components was observed to be low HDL-cholesterol, elevated triglycerides, and central obesity, respectively. Conclusions: The alarming increase in pediatric MetS among Arab children and adolescents over a short timespan have significant clinical and economic implications if not addressed immediately. Health policy makers should implement lifestyle interventions aimed at high-risk children or overweight youths.

## 1. Introduction

The global industrialization has resulted in significant transitions worldwide in terms of lifestyle and eating behaviors of individuals [1,2]. These changes included increased physical inactivity and sedentary behavior coupled with higher consumption of energy-dense diet and sugary beverages in highly urban areas [3,4]. Consequently, the worldwide prevalence of obesity has continuously increased [5,6], which led to a steady increase in the prevalence of cardiovascular diseases (CVDs) and type 2 diabetes mellitus (T2DM) [7,8]. This phenomenon is not limited to the adult population. In fact, it has been estimated that the prevalence of obesity or overweight among school children (aged 5–17 years) was about 10% worldwide [5]. Saudi Arabia is not immune to the widespread increase in global childhood obesity. In fact, in 2010 alone, the overall prevalence of overweight, obesity, and severe obesity among children and adolescents was 23.1%, 9.3%, and 2%, respectively, based on the analysis of 19,317 children and adolescents aged 5–18 years (50.8% boys) [9]. These figures increased in 2014, with a reported prevalence of overweight and obesity in Saudi boys (aged 6–13 years) of 7.3% and 17.4%, respectively, while in girls, it was 12.4% and 20.9%, respectively [10]. According to the World Health Organization (WHO) report, the prevalence of obesity and overweight in Saudi children aged 5–19 again worsened in 2016 (17.4% and 35.6%, respectively) [11]. A recent study in Riyadh region on 7930 children (aged 6–16 years) indicated that the prevalence of overweight and obesity was 12% and 18.4%, respectively, while in girls it was 14.2% and 18%, respectively [12].

Aside from obesity, another unfavorable health consequence of urbanization is metabolic syndrome (MetS). MetS is the clustering of cardiometabolic risk factors, which predisposes an individual to CVD and T2DM. These risk factors include insulin resistance (IR), central obesity, low levels of high-density lipoproteins (HDL), hypertension, and elevated triglycerides (TG) [13]. Currently, MetS is considered a complex condition and various features are still not fully understood [8,13,14,15]. Childhood MetS and cardiometabolic abnormalities are increasingly becoming more common, as well [2,16,17]. The early clustering of MetS components is alarming, considering that these components may persist into adulthood, which significantly puts an individual at the risk of future T2DM and CVD [18]. Numerous studies in children and adolescents have demonstrated that the persistence of childhood MetS is associated with T2DM and CVD in adulthood [19]. Therefore, the prevention of childhood MetS is of great clinical importance in order to improve the health outcomes throughout the adolescence stage, leading to a reduction in the incidence of CVD and T2DM in adulthood. In a 2013 systematic review describing the worldwide epidemiology of MetS in children, authors have reported a median prevalence of 3.3% (0–19.2%), in which the MetS prevalence in overweight children was 11.9% (2.8–29.3%) and 29.2% (10.0–66.0%) in obese children [17]. In Saudi Arabia, as of 2010, the over-all prevalence of MetS among 1231 Saudi students aged 10–18 years in Riyadh was 9.4% [confidence interval (CI) 7.8–11.1] [20]. A low HDL-cholesterol was the most prevalent of all MetS risk factors, affecting 86% (CI 85.0–88.6) and hypertriglyceridemia, the second most prevalent, affecting 33% (CI 30.6–35.8) of the participants [20]. Aside from the increased sedentary lifestyle and over nutrition, other factors that may have contributed to the high prevalence of cardiometabolic disorders in childhood MetS included family history [21], vitamin D deficiency [22,23], and increased visceral adiposity [24].

Given the increasing trends in childhood obesity worldwide, it is worthwhile to investigate whether other cardiometabolic risks, such as MetS, are becoming more common, especially in high-risk and understudied ethnic groups, such as the Arab youth. The present study aims to fill this gap, and assess the trends in the prevalence of MetS among Arab children and adolescents (aged 12–18 years) from different schools in Riyadh city, Saudi Arabia by comparing data collected from previous cohorts in 2010, 2015, and 2019.

## 2. Materials and Methods

### 2.1. Participants and Data Sources

This cross-sectional study included a total of 7985 apparently healthy Saudi children and adolescents, aged 12–18 years from three different cohorts. The participants were recruited randomly from different households and governmental schools across the city of Riyadh, Saudi Arabia. Prior to the study, each student submitted an assent form. In addition, parents signed a written consent, as well as answered a general questionnaire containing past and present demographic and medical history.

**Figure 1:**
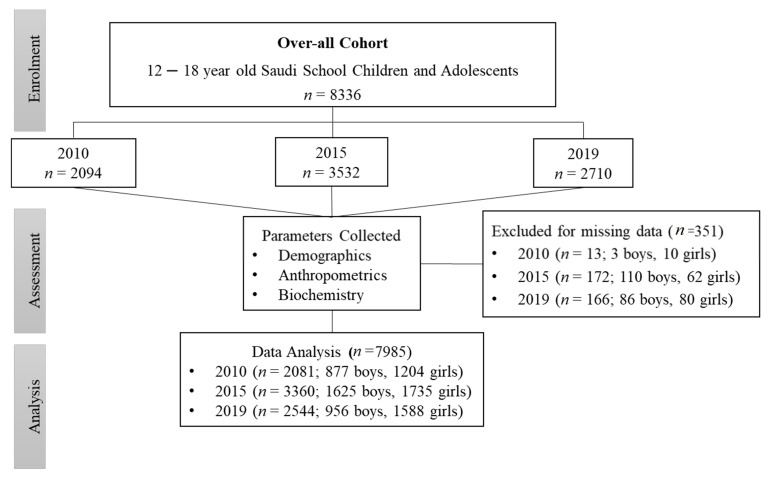
Flowchart of the participants.

Participants’ data were obtained from two projects as follows: Data from 2010 were collected from the Riyadh Cohort database [20], where participants were invited door-to-door from different households, and assessments were performed at the nearest primary care center. Data from 2015 were collected from the Vitamin D Schools Project database, a collaborative study between the Prince Mutaib Chair for Biomarkers of Osteoporosis (PMCO) in King Saud University (KSU) and the Ministry of Education in Saudi Arabia involving 34 schools. The project was registered in the Saudi Food and Drug Administration (SFDA) clinical trial registry (SCTR no. 16012402). The detailed structure of the 2015 study participants was described elsewhere [25]. The 2019 participants’ data were collected from the diabetes schools project, a collaborative study between the Chair for Biomarkers of Chronic Diseases (CBCD) in King Saud University (KSU) and the Saudi Diabetes Charity Association, Riyadh, Saudi Arabia, also involving the same schools. Ethical approval was obtained from King Saud University Medical City (KSUMC) (no. E19-4239). Figure 1 shows the flowchart of the participants.

### 2.2. Anthropometric Measurements

The participants were instructed to come to their respective school in a 10-h overnight fasting state. Anthropometric measurements were collected by trained nurses, including weight (cm), height (cm), body mass index BMI (kg/m^2^), hip (cm), and waist (cm) circumferences. Systolic and diastolic blood pressures were measured as the average of two readings with a 15-min interval, using pediatric cut-offs appropriate for children’s sizes.

### 2.3. Biochemical Analyses

Fasting blood samples were collected by trained nurses. Biochemical analyses, including fasting blood glucose, high-density lipoprotein cholesterol (HDL-C), total cholesterol (TC), and triglycerides were measured using a standard routine laboratory analysis (Konelab, Finland). The LDL cholesterol (LDL-C) was calculated using the Friedwald equation.

### 2.4. Definition of MetS

MetS was defined according to the criteria of de Ferranti et al. [26] for comparison purposes, which was the same criteria used in the 2010 database. The choice of MetS definition was based on the original criteria used in 2010, as well as its applicability for mass screening, lack of prerequisite risk factors, and its applicability to capture a higher yield of high-risk children as compared to the other definitions [27]. Participants with three or more of the following MetS component were defined as having MetS.

1. Hypertriglyceridemia ≥ 1.1 mmol/L.
2. Low HDL-cholesterol < 1.3 mmol/L (boys aged 15–19 years, <1.7 mmol/L).
3. High fasting glucose ≥ 6.1 mmol/L.
4. Central obesity: >75th percentile for age and gender.
5. Hypertension: >90th percentile for age, gender, and height.

### 2.5. Data Analysis

Data were analyzed using SPSS (version 22 Chicago, IL, USA). Continuous data were presented as mean ± standard deviation (SD) for normal variables and non-Gaussian variables were presented in median (1st and 3rd) percentiles. Categorical data were presented as frequencies, percentages (%), and 95% confidence interval. All continuous variables were checked for normality using the Kolmogorov-Smirnov test. Non-Gaussian variables were log-transformed prior to the parametric analysis. The independent *t*-test and Mann Whitney U were used to compare the mean and median differences in Gaussian and Non-Gaussian variables. The change response rate in MetS and its components were analyzed by two proportions of the population on a gender basis. *p*-value < 0.05 was considered statistically significant.

## 3. Results

Table 1 shows the differences in cardiometabolic characteristics of boys and girls at different time points. In the 2010 cohort, boys had significantly higher BMI, waist, hips, and total cholesterol as compared to girls, while the girls had significantly higher HDL-cholesterol levels than boys (*p*-values < 0.05). In the 2015 cohort, girls had significantly higher waist, hips, diastolic blood pressure, and HDL-cholesterol than boys (*p*-values < 0.05). On the other hand, the boys had significantly higher systolic blood pressure and glucose than girls (*p*-values < 0.05). Finally, in the 2019 cohort, the boys had significantly higher BMI, systolic blood pressure, glucose, and HDL-cholesterol than girls, while the girls were significantly older and had higher waist, hips, diastolic blood pressure, triglycerides, and total cholesterol (*p*-values < 0.05) (Table 1).

Table 2 shows the prevalence of MetS in different time points. In 2010, the prevalence of MetS was 11.8%, which increased to 20.1% in 2015 and again in 2019 to 20.6%. Stratified according to sex, the prevalence of MetS in boys was only 7.8% in 2010, which increased to 23% in 2015 and again to 25.3% in 2019. The increase in the prevalence of MetS was also observed in girls, but this increase was modest with 14.8% in 2010, to 17.5% in 2015 to 17.7% in 2019.

Table 3 shows the prevalence in the different components of MetS according to the different time points. Central obesity increased significantly from 17.1% in 2010 to 22.8% in 2015 and 26.5% in 2019 (*p* < 0.05). The same increase was observed in the prevalence of elevated blood pressure and low-HDL cholesterol (*p*-values < 0.05). A sharp increase was noted in the prevalence of elevated blood glucose from 5.1% in 2010 to 9% in 2015, as well as elevated triglycerides from 17.1% in 2010 to 43.4% in 2015. Finally, a steady increase in the prevalence of obesity was noted from 8.3% in 2010 to 15.1% in 2015 and finally 17.3% in 2019.

Percentage changes in the prevalence of MetS and its components in boys and girls from 2010 to 2019 are shown in Table 4. In boys, there was a 17.5% increase in the prevalence of MetS and this was significantly higher than girls, which was only 2.9%. Boys also had a significantly higher percentage increase than girls in all MetS components, the highest increase of which was observed in low HDL-cholesterol and elevated triglycerides (*p*-values < 0.01).

**Figure 2:**
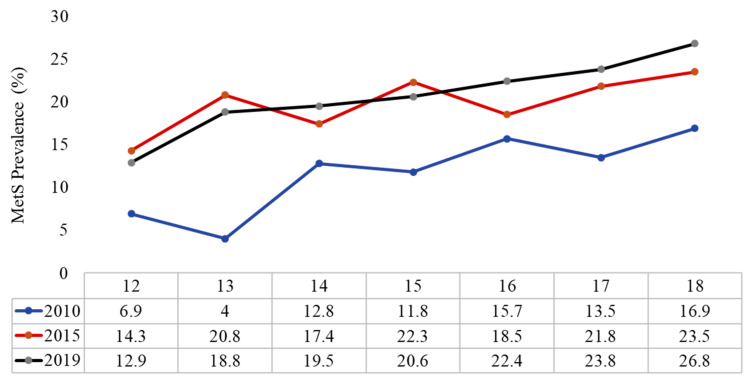
Prevalence of MetS according to the age groups overtime.

**Figure 3:**
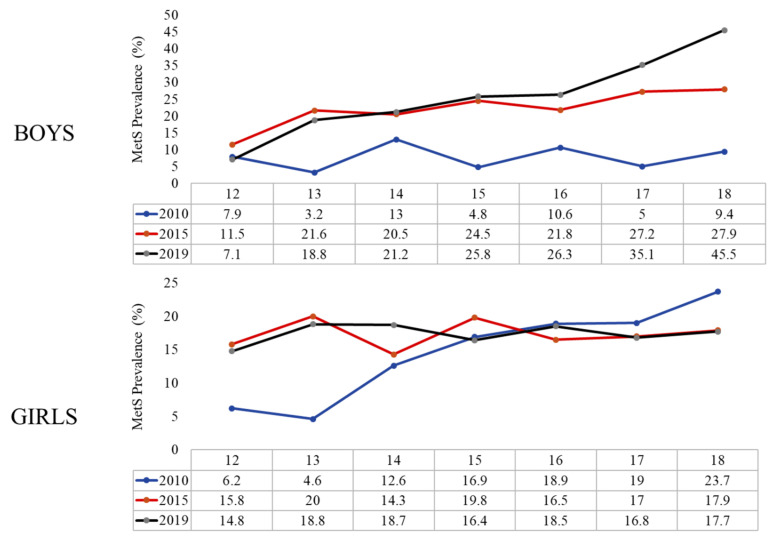
Prevalence of MetS according to the age groups in boys and girls overtime.

Finally, Figure 2 shows the increasing prevalence of MetS according to age in different time points. Stratification according to sex showed that the increasing prevalence was evident in both boys and girls, but it was more prominent in boys (Figure 3, Supplementary Table S1). The prevalence of the different MetS components in boys and girls is shown in Supplementary Table S2.

## 4. Discussion

The present cross-sectional study evaluated trends in the prevalence of MetS among Saudi children and adolescents from data collected in three time points (2010, 2015, and 2019), using the criteria proposed by de Ferranti et al. [26]. The present results showed increasing trends in the prevalence of MetS among Saudi children regardless of sex, with an increasing prevalence by more than 2-fold in 2019 compared to 2010, 20.6% vs. 9.4%, respectively.

Compared to the other countries using the same criteria, Asghari et al. assessed 1424 Iranian adolescents aged 11–18 (55.2% female) and found the MetS prevalence to be 26.4% [28]. Among East Asians, a recent Chinse study on 2761 adolescents aged 15–19 years reported that the overall prevalence of MetS was 3.7% [29]. In Hispanic groups, Ramirez et al. found that the prevalence of MetS was 11.0% in 675 children and 1247 adolescents attending public schools (54.4% girls; age range 9–17.9 years) [30]. Within Saudi Arabia, Bahathiq in 2018 reported a prevalence of 17.1% among 1356 school girls (6 to 18 years) from the Makkah area [31]. In 2014, Al-Hussein et al. using six different definitions for MetS, reported a prevalence ranging from 2% to 18% among 2149 Saudi schools’ boys and girls (aged 6 to 17 years) in Riyadh [32]. The discrepancy can be attributed to the relatively older age in our study population and the children were of a pubertal age. Comparing the present results to other Arab countries, our results are higher than what was reported in the United Arab Emirates at 3.7% in a total of 596 students (308 boys and 288 girls) aged 10 to 15.9 years [33]. This difference can be attributed to the different MetS definitions that were used.

Our results showed differences in the prevalence of MetS between boys and girls. The MetS prevalence was higher in boys than girls in both the 2015 and 2019 data. This disparity could be due to the significantly higher rates of low HDL-C levels and elevated triglycerides in boys than in girls. This is in agreement with the study in UAE using IDF criteria [33]. In contrast, Tandon et al. found a lower prevalence of MetS in boys compared to girls [34]. A low HDL-C was the most common MetS component, followed by elevated triglycerides, which is consistent with our previous 2010 results [20]. At the same time, Khashayar et al. on a nationally representative sample of 5738 Iranian adolescent aged 10–18 years, found that having a low HDL-C was the most common MetS component representing 43.2% of all the study population [35].

Finally, it is important to address the possible reasons for the sharp increase in pediatric MetS over time among Arab youth. Although not explicitly measured in the present study, one reason is the high prevalence of physical inactivity among Arab youth. In 2004, the reported prevalence of physical inactivity was already 57.1% in children and 71.1% in adolescents [36]. Sedentary behavior and low physical activity seem to have worsened over time, with data in 2017 involving 1133 participants aged 14–19 revealing a very high prevalence of excessive screen time (84.6%), with 82.4% falling below the recommended physical activity [37]. The sharp rise of pediatric MetS in the present study from 2010–2015 coincided with the smartphone revolution of the 2010s. By 2013, mobile phone ownership among Saudis 8–18 years old was 87%, of which 71% were smartphones (GSMA) [38]. This early exposure to digital technology has led to excessive screentime and addiction to gaming and smartphone which promotes stress [39,40], as well as an unhealthy lifestyle that has been observed elsewhere under similar conditions [41,42,43], aggravating the prevalence of pediatric MetS in Saudi youth. Of note, the prevalence of pediatric MetS has in some way plateaued in females from 2015–2019, and this could be due to the recent socio-political changes in Saudi Arabia, which has removed traditional barriers and opened the doors for females to engage in sports and increase social mobility [44].

Our study has some limitations: First, we did not assess the socioeconomic status of the participants’ families, physical activity, dietary intakes, and other extraneous influences that could affect the MetS risk factors. These factors would have added clarity to the increasing trend if they were measured in different time points. Furthermore, the observational design of the study cannot infer any causality.

## 5. Conclusions

The present study highlights the alarming prevalence rate of MetS among Saudi children and adolescents which has worsened overtime, particularly in boys. At the family level, parental education is needed for restrictions on games and smart phone use among children. Furthermore, more involvement from the government to address pediatric MetS among Saudi youth is needed, in order to attain meaningful and favorable outcomes.

## Figures and Tables

**Table 1:** Cardiometabolic characteristics of boys and girls in 2010, 2015, and 2019.

Parameters	2010		2015		2019	
	Boys	Girls	Boys	Girls	Boys	Girls
*n*	877	1204	1625	1735	956	1588
Age (years)	15.1 ± 1.9	15.1 ± 1.9	14.9 ± 1.8	14.8 ± 1.9	14.9 ± 1.6	14.7 ± 1.7 **
BMI (kg/m^2^)	22.5 ± 5.4	21.6 ± 5.4 **	22.9 ± 6.1	22.9 ± 5.2	23.6 ± 6.9	22.7 ± 6.1 **
BMI z-score	0.11 ± 0.9	−0.07 ± 0.99 **	−0.004 ± 1.1	0.003 ± 0.9	0.09 ± 1.1	−0.06 ± 0.9 **
Waist (cm)	70.6 ± 15.1	68.2 ± 19.2 *	65.2 ± 15.1	72.2 ± 10.9 **	67.8 ± 14.8	72.5 ± 11.6 **
Hips (cm)	84.7 ± 17.2	77.5 ± 21.0 **	74.0 ± 27.2	94.1 ± 11.9 **	76.9 ± 27.4	92.1 ± 12.6 **
Systolic BP (mmHg)	107.4 ± 10.2	108.4 ± 10.9	123.0 ± 15.0	116.9 ± 13.8 **	119.5 ± 15.6	117.8 ± 18.5 *
Diastolic BP (mmHg)	69.9 ± 7.3	70.2 ± 7.7	69.8 ± 13.5	71.3 ± 11.4 **	67.3 ± 11.0	75.4 ± 14.1 **
Glucose (mmol/L)	5.2 ± 1.4	5.3 ± 1.3	5.3 ± 1.2	5.1 ± 0.9 **	5.3 ± 1.2	5.2 ± 1.2 **
Triglycerides (mmol/L)	1.1 ± 0.7	1.1 ± 0.6	1.2 ± 0.7	1.1 ± 0.5 **	1.2 ± 0.6	1.1 ± 0.5 **
Total Cholesterol (mmol/L)	4.3 ± 0.9	4.01 ± 0.8 **	3.9 ± 0.9	4.0 ± 1.1	4.3 ± 0.8	4.4 ± 0.7 **
HDL-Cholesterol (mmol/L)	0.94 ± 0.3	0.88 ± 0.3 **	0.97 ± 0.3	1.1 ± 0.3 **	0.99 ± 0.2	0.98 ± 0.3 *

Note: Data presented mean ± SD, * denotes significance at the 0.05 and ** at 0.01 level.

**Table 2:** Prevalence of MetS in the 2010, 2015, and 2019 cohort.

Parameters	Year								
	2010 (*n* = 2081)			2015 (*n* = 3360)			2019 (*n* = 2544)		
	*n*	MetS %	CI 95%	*n*	MetS %	CI 95%	*n*	MetS %	CI 95%
Overall	246	11.8	10.5–13.3	676	20.1	18.8–21.5	523	20.6	19.0–22.2
Boys	68	7.8	6.1–9.7	373	23	20.9–25.1	242	25.3	22.6–28.2
Girls	178	14.8	12.8–16.9	303	17.5	15.7–19.3	281	17.7	15.8–19.7

Note: CI: Confidence interval.

**Table 3:** Overall prevalence of MetS components in the 2010, 2015, and 2019 cohort.

Year	2010	2015	2019
*n*	2081	3360	2544
MetS Components			
Central Obesity (cm)	355 (17.1, 12.3–15.3)	765 (22.8, 21.4–24.2)	676 (26.5, 24.9–28.3) **
Elevated Blood Pressure (mmHg)	286 (13.7, 12.3–15.3)	534 (15.9, 14.7–17.2)	465 (18.3, 16.8–19.8) **
Elevated Glucose (mmol/L)	107 (5.1, 4.2–6.2)	304 (9.0, 8.1–10.1)	123 (4.8, 4.0–5.7) **
Low HDL-Cholesterol (mmol/L)	932 (44.8, 42.6–47.0)	2606 (77.6, 76.1–79.0)	2214 (86.8, 85.5–88) **
Elevated Triglycerides (mmol/L)	355 (17.1, 15.5–18.7)	1459 (43.4, 41.7–45.1)	945 (37.0, 35.1–38.9) **
Obesity (kg/m^2^)			
Normal	1637 (78.7, 76.8–80.4)	2097 (62.4, 60.7–64.1)	1580 (62.1, 60.2–64.0)
Overweight	272 (13.1, 11.7–14.6)	757 (22.5, 21.1–24.0)	525 (20.6, 19.1–22.3)
Obese	172 (8.3, 7.1–9.5)	506 (15.1, 13.9–16.3)	439 (17.3, 15.8–18.8)

Note: Data presented as *n* (%, 95% CI). ** represented the *p*-value, which is significant at the 0.05 and 0.01 level from 2015 to 2020, respectively for overall, boys, and girls.

**Table 4:** Prevalence of MetS components in the 2010, 2015, and 2019 cohort.

MetS Components	Percentage Change % (2010 vs. 2019)		*p*-Value
	Boys	Girls	
MetS	17.5	2.9	<0.001
Central Obesity	14.1	6.2	<0.001
Elevated Blood Pressure	4.9	4.0	<0.001
High Glucose	2.3	-2.0	<0.001
Low HDL-Cholesterol	58.2	31.2	<0.001
Elevated Triglycerides	30.1	13.5	<0.001

Note: Data presented as percentage (%). Significant at *p* < 0.05.

## Data Availability

The data presented in this study are available on request from the corresponding author. The data are not publicly available due to the privacy protection.

## Supplementary Materials

The following are available online at https://www.mdpi.com/article/10.3390/children8121129/s1. Table S1: Prevalence of MetS in boys and girls according to age overtime. Table S2: Prevalence of MetS components in 2010, 2015, and 2019 in boys and girls.
