# Supplementary material for: Increasing Prevalence of Pediatric Metabolic Syndrome and Its Components among Arab Youth: A Time-Series Study from 2010–2019
Source: Children (Basel). 2021 Dec 3;8(12):1129. doi: 10.3390/children8121129 (PMC8700288; doi:10.3390/children8121129)
Supplement: Supplementary file 1 [file children-08-01129-s001.zip › children-1472149-supplementary.pdf]

Supplementary Table S1. Prevalence of MetS in Boy and Girls according to Age overtime.

**Year**	**2010**		**2015**		**2019**	
	**Boys**	**Girls**	**Boys**	**Girls**	**Boys**	**Girls**
n	877	1204	1625	1735	956	1588
Age						
12	9 (7.9, 3.7–14.5)	9 (6.2, 2.9–11.4)	10 (11.5, 5.7–20.1)	26 (15.8, 10.6–22.2)	3 (7.1, 1.5–19.5)	19 (14.8, 9.2–22.2)
13	4 (3.2, 0.9–8.1)	8 (4.6, 2.0–8.9)	82 (21.6, 17.5–26.1)	74 (20.0, 16.0–24.4)	30 (18.8, 1.3–25.7)	63 (18.8, 14.8–23.4)
14	16 (13.0, 7.6–20.3)	21 (12.6, 8.0–18.6)	61 (20.5, 16.0–25.5)	42 (14.3, 10.5–18.8)	36 (21.2, 15.3–28.1)	67 (18.7, 14.8–23.1)
15	6 (4.8, 1.8–10.2)	29 (16.9, 11.6–23.3)	69 (24.5, 19.6–29.9)	48 (19.8, 14.9–25.3)	57 (25.8, 20.2–32.1)	45 (16.4, 12.2–21.3)
16	14 (10.6, 5.9–17.2)	39 (18.9, 13.8–25.0)	31 (21.8, 15.3–29.5)	40 (16.5, 12.1–21.8)	51 (26.3, 20.2–33.1)	36 (18.5, 13.3–24.6)
17	6 (5.0, 1.9–10.6)	35 (19.0, 13.6–25.4)	63 (27.2, 21.5–33.4)	44 (17.0, 12.6–22.1)	40 (35.1, 26.4–44.6)	31 (16.8, 11.7–23.1)
18	13 (9.4, 5.1–15.5)	37 (23.7, 17.3–31.2)	57 (27.9, 21.9–34.6)	29 (17.9, 12.3–24.7)	25 (45.5, 32.0–59.4)	20 (17.7, 11.2–26.0)

Note: Data presented *n* (%, 95%CI).

Supplementary Table S2. Prevalence of MetS components in 2010, 2015 and 2019 in Boys and Girls.

**Year**	**2010**		2015		2019	
**MetS Components**	**Boys**	**Girls**	Boys	Girls	Boys	Girls
Central Obesity (cm)	109 (12.4)	246 (20.4)	323 (19.9)	442 (25.5)	253 (26.5) **	423 (26.6)
Elevated Blood Pressure (mmHg)	101 (11.5)	185 (15.4)	301 (18.5)	233 (13.4)	157 (16.4)	308 (19.4) **
Elevated Glucose (mmol/L)	35 (4.0)	72 (6.0)	183 (11.3)	121 (7.0)	60 (6.3) **	63 (4.0) **
Low HDL-Cholesterol (mmol/L)	316 (36.0)	616 (51.2)	1409 (86.7)	1197 (69.0)	901 (94.2) **	1308 (82.4) **
Elevated Triglycerides (mmol/L)	124 (14.1)	231 (19.2)	790 (48.6)	669 (38.6)	423 (44.2) *	519 (32.7) **
Obesity (kg/m^2)						
Normal	666 (75.9)	971 (80.6)	999 (61.5)	1098 (63.3)	544 (56.9)	1036 (65.2)
Overweight	126 (14.4)	146 (12.1)	346 (21.3)	411 (23.7)	188 (19.7)	337 (21.2)
Obese	85 (9.7)	87 (7.2)	280 (17.2)	226 (13.0)	224 (23.4)	215 13.5) **

Note: Data presented *n* (%); * and ** denote *p*-values at 0.05 and 0.01 level from 2015 to 2019 respectively for overall, boys and girls.
